# Prevalence of Poisoned Google Search Results of Erectile Dysfunction Medications Redirecting to Illegal Internet Pharmacies: Data Analysis Study

**DOI:** 10.2196/38957

**Published:** 2022-11-08

**Authors:** András Fittler, Péter Paczolai, Amir Reza Ashraf, Amir Pourhashemi, Péter Iványi

**Affiliations:** 1 Department of Pharmaceutics Faculty of Pharmacy University of Pécs Pécs Hungary; 2 Department of Systems and Software Technologies Faculty of Engineering and Information Technology University of Pécs Pécs Hungary

**Keywords:** internet pharmacies, search engine redirection, compromised websites, illegal medicines, patient safety, Europe, erectile dysfunction medications

## Abstract

**Background:**

Illegal online pharmacies function as affiliate networks, in which search engine results pages (SERPs) are poisoned by several links redirecting site visitors to unlicensed drug distribution pages upon clicking on the link of a legitimate, yet irrelevant domain. This unfair online marketing practice is commonly referred to as search redirection attack, a most frequently used technique in the online illegal pharmaceutical marketplace.

**Objective:**

This study is meant to describe the mechanism of search redirection attacks in Google search results in relation to erectile dysfunction medications in European countries and also to determine the local and global scales of this problem.

**Methods:**

The search engine query results regarding 4 erectile dysfunction medications were documented using Google. The search expressions were “active ingredient” and “buy” in the language of 12 European countries, including Hungary. The final destination website legitimacy was checked at LegitScript, and the estimated number of monthly unique visitors was obtained from SEMrush traffic analytics. Compromised links leading to international illegal medicinal product vendors via redirection were analyzed using Gephi graph visualization software.

**Results:**

Compromised links redirecting to active online pharmacies were present in search query results of all evaluated countries. The prevalence was highest in Spain (62/160, 38.8%), Hungary (52/160, 32.5%), Italy (46/160, 28.8%), and France (37/160, 23.1%), whereas the lowest was in Finland (12/160, 7.5%), Croatia (10/160, 6.3%), and Bulgaria (2/160, 1.3%), as per data recorded in November 2020. A decrease in the number of compromised sites linking visitors to illegitimate medicine sellers was observed in the Hungarian data set between 2019 and 2021, from 41% (33/80) to 5% (4/80), respectively. Out of 1920 search results in the international sample, 380 (19.79%) search query results were compromised, with the majority (n=342, 90%) of links redirecting individuals to 73 international illegal medicinal product vendors. Most of these illegal online pharmacies (41/73, 56%) received only 1 or 2 compromised links, whereas the top 3 domains with the highest in-degree link value received more than one-third of all incoming links. Traffic analysis of 35 pharmacy specific domains, accessible via compromised links in search engine queries, showed a total of 473,118 unique visitors in November 2020.

**Conclusions:**

Although the number of compromised links in SERPs has shown a decreasing tendency in Hungary, an analysis of the European search query data set points to the global significance of search engine poisoning. Our research illustrates that search engine poisoning is a constant threat, as illegitimate affiliate networks continue to flourish while uncoordinated interventions by authorities and individual stakeholders remain insufficient. Ultimately, without a dedicated and comprehensive effort on the part of search engine providers for effectively monitoring and moderating SERPs, they may never be entirely free of compromised links leading to illegal online pharmacy networks.

## Introduction

### Background

The inherent practicality and convenience of online shopping are proving increasingly influential in consumer’s behavior worldwide. Based on the 2020 e-commerce statistics published by Eurostat [1], 89% of all European Union (EU) citizens used the internet within the last 12 months, and 65% of individuals made an online purchase in the same period. Nonprescription medicine or dietary supplements accounted for 28% of these transactions, demonstrating consumers’ growing trust in online health- and well-being–related purchases [1]. A large-scale study [2] of changes in information-seeking behavior showed that the most frequently mentioned content is “product information” and “purchase” (30% of all responses in 1997 and 2019), followed by “Health” (18% of all responses in 1997 and 19% in 2019) [2]. Notably, user behavior had been remarkably consistent in the span of 22 years [2].

The use of internet pharmacies and the number of individuals obtaining medications and various health products online are increasing [2]. Several advantages including perceived anonymity, cost savings, and convenience motivate individuals to purchase medications online [3]. Furthermore, the lack of a valid prescription required by legal online and offline vendors is a strong driving force toward illegal online drug purchases [3]. However, several patient safety risks are linked to the procurement of medicines outside the traditional supply chain, including questionable sourcing, poor product quality, substandard and falsified medicines, improper storage, and transportation [4]. Risks are augmented by rogue internet pharmacies considered as a primary source of substandard and falsified medical products in developed countries [5-7].

The widespread availability of search engines and increased public interest in obtaining medicines online imply a major dilemma, whether consumers aiming to purchase medications from the internet are starting their online activity from relevant web pages (eg, a national authority website), or simply searching using their search engine of choice. Most likely the latter is the case. Search engines refer consumers to relevant online resources quickly. Their significance is illustrated by the fact that most trackable website traffic originates from search engines [8], and typically from Google as this platform is handling more than 90% of search queries worldwide. Online distributors choose to use several digital marketing techniques to attract customers via search engines. Website operators apply various search engine optimization (SEO) techniques to improve the visibility of their websites, a practice that is accepted and supported by search engines [9]. SEO is a complex and time-consuming procedure, especially in the international marketplace in which country- and language-specific optimization is required to reach a high-ranking position among organic query results.

For illegal medicine sellers, conventional SEO is neither cost- nor time-effective, as they are constantly threatened with regulatory closure [10]. Furthermore, paid advertisements offering prescription drugs without a prescription by unauthorized pharmacies cannot appear in any of the major paid search advertising services [11,12]. Therefore, alternative dishonest digital marketing methods including web spamming, forum abuse, and additional “black hat” SEO techniques are used by illegal drug distribution websites to promote their links in the unpaid search engine results pages (SERPs) to gain favorable search engine rankings [13,14].

As a result, the user’s query on a search engine may contain both “normal” domains (ie, those related to the query) and “compromised/deceptive” domains (ie, ones that are unrelated to the query). The latter domains are promoted in the rank using “black hat” SEO methods, undermining the value proposition of search engines, as search results are presented with deceptive views of a website with inflated relevance to selected search terms. Individuals (search engine users) are referred to low-quality content or malicious websites when clicking on a deceptive search result. Consequently, the deceptive web pages practically “poison” the search result; therefore, this technique is termed as “search engine poisoning” or “search redirection attack” [9,15].

Manipulation of search results for erectile dysfunction medications was published nearly a decade ago by Leontiadis et al [15,16] and Wang et al [17]. Sildenafil was the first commercially available phosphodiesterase type 5 (PDE5) inhibitor available since 1998, followed by vardenafil, tadalafil, and avanafil [18]. Increasing prevalence of erectile dysfunction and widespread use of PDE5 inhibitors as the first-line oral treatment worldwide [19] have resulted in growing demand, which illegal online vendors have been taking advantage of [20].

### Objectives

The major aim of our study is to introduce the relatively unknown but significant and persistent issue of poisoning of search engine results (SERs) of erectile dysfunction medications in European countries. Furthermore, the study is meant to measure the scale of the problem and illustrate the redirection networks referring users (patients) to illegal internet pharmacies. Public health significance of the problem is illustrated by the estimation of the likelihood of consumers clicking on poisoned search results and the number of monthly visitors redirected to illicit pharmacy networks. Our utmost aim is to warn the general public and raise the awareness of authorities and law enforcement agencies, thus facilitating long-awaited countermeasures.

## Methods

### Mechanism of Search Engine Poisoning and Redirection

**Figure 1:**
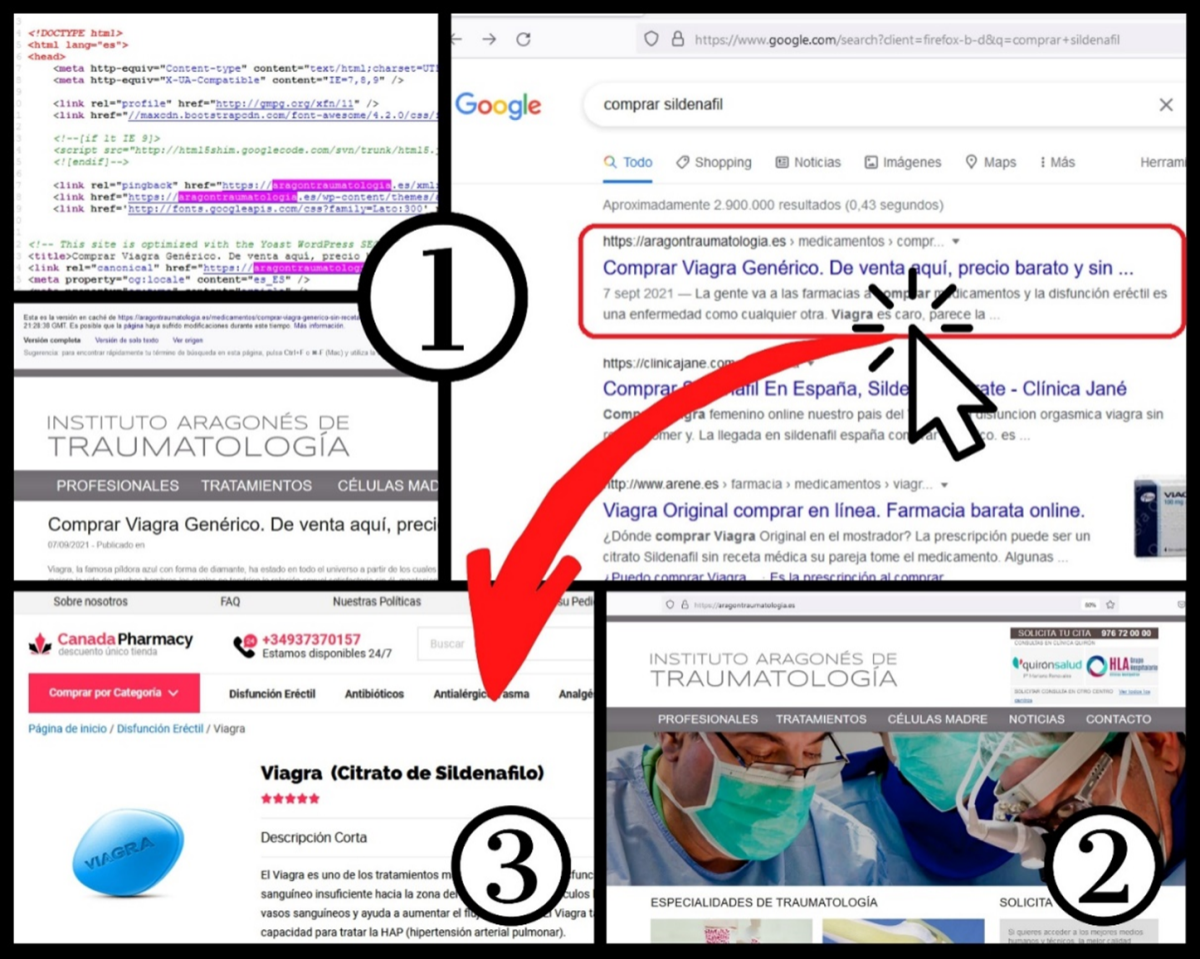
Illustrative figure of how users pass through a redirection chain from the search result page to the final destination illegal online pharmacy website.

A search engine poisoning attack begins with an attacker hacking into a vulnerable web page. Common targets are outdated, vulnerable, or complex content management and blogging systems (eg, WordPress; see Figure 1, part 1). Once the attacker has access to the system, a new code is injected, and the hacked website will “interrupt” all incoming HTTP requests to the original web page and respond to these requests differently from the original operation [15]. Typically, users are redirected through a redirection chain, consisting of intermediate pages to a final page. The destination is the illegal pharmacy website most users are unwillingly visiting. However, users do not see the original content of the compromised website after clicking on the search results, because they are presented with the unwanted final page, as hacked websites redirect the web browsers within milliseconds. Redirection attacks—identifiable in various search engines such as Google, Bing, and Yahoo!—disregard term relevance constraints and target search terms of the actual search; however, at the same time, the original content of the hacked website (domain) becomes irrelevant to the search terms used (see Figure 1, part 2).

In the case of search engine poisoning attack, it is important that compromised websites look differently, depending on the visitor, due to the so-called cloaking method [13]. The original content stuffed with keywords and links to increase page rank is shown to the automated agent/crawler (eg, Google bot), meanwhile the redirected illegitimate online vendor is displayed to the customer (see Figure 1, part 3) [16]. Currently no efficient technique capable of identifying all spam web pages is available [13]. Because of the cloaking method used by the illegitimate pharmacy operators, the automation of the content evaluation of SERs is difficult and precise detection requires manual assessment or checking.

### Obtaining and Evaluating SERs in National and International Data Sets

Search engine query results and links were documented and manually evaluated to simulate and evaluate what consumers see while browsing. Manual data acquisition was necessary as automatic search queries are prohibited by search engine providers and cloaking is difficult to identify automatically. The focus of the research was on erectile dysfunction medications as a popular category affected by illegal online trade and potential source of substandard and falsified medicinal products [20,21]. Consequently, the search queries represent purchase intent (buying prescription medications online), rather than informative types of search (looking for product information). The 4 primary active pharmaceutical ingredients (APIs), sildenafil, tadalafil, vardenafil, and avanafil, were searched for using Google, the most popular search engine. Country-specific data were obtained by individualizing national search using the search terms of the “API” and the “buy” words in the language of the given country (eg, “comprar sildenafil” for Spain). Furthermore, search settings in Google have been adjusted to the preferred region. To track the evolution of the phenomena, the first 20 organic SERs were evaluated during 3 consecutive years: August and October 2019, August 2020, and November 2021 for the national data set. Meanwhile, the first 40 SERs were included in the international data set evaluated in November 2020. Accordingly, we conducted our research on 2 data sets: a long-term evaluation of Hungarian SERs and an international sample in Hungary and an additional 11 other countries (Bulgaria, Croatia, Estonia, Finland, France, Greece, Italy, Romania, Spain, Sweden, and United Kingdom) from different regions of Europe. As most (88%) users click on results appearing in the top 10 SER positions [22], by documenting the top 20 results we consider our findings representative for online queries at the time of evaluation. SER links of websites offering medicinal products for sale were included for evaluation; nonrelevant query results were excluded from our evaluation.

The documented search result data included date, country, search language, API, search phrase, URL and domain name, SER ranking, destination website URL for redirections, and website category. Two figures were used to describe the significance of the phenomena regarding search engine redirection attacks in SERs: (1) prevalence of hacked links in SERPs and (2) cumulative click-through rate (CTR). Both measures correlate with the likelihood of users—intentionally or unintentionally—visiting illegal pharmacies. Prevalence is calculated by dividing the number of infected links by the total number of evaluated links in SERPs. Based on Google’s organic search ranking, CTR is a probability value of clicking on a given link assigned to each measured SER position. On the first page of the search (Google) result, 1-10 CTR per ranking values were determined based on the analysis by Sistrix [22], while further CTRs for 11-40 SER positions were computed with the equation of the exponential trend line connecting the first 1-10 SERP datapoints (y=26,76e^–0.258x^, where y is the predicted CTR and x is the SER rank; *R*^2^=0.927). Cumulative CTRs express the sum of CTR values regarding all documented positions in SERPs.

Compromised sites redirecting to international illegal medicine retailers have been classified into 3 categories referencing the redirection’s life cycle based on Leontiadis et al [16]. First, the compromised site is likely a future redirect (hacked website content with or without links; however, no automatic redirection is yet observed). Second, active redirection to an international illegal medicinal product vendor via a compromised site. Lastly, inactive redirection, that is, sites used to be redirecting, but no longer redirecting, because they are not accessible at the time of evaluation, displaying 404 error code, or similar.

### Graph Visualization, Legitimacy, and Traffic Analysis Regarding Destination Websites

Compromised SERP links leading to international illegal medicinal product vendors via redirection (active links) were evaluated and networks have been generated with Gephi [23], an open-source graph visualization and analysis tool. The national and international data sets were visualized as directed graphs illustrating the source and destination website domains. Multiple links from the same domain accounted for increased weight of the edge. The average degree (average number of edges per node in the graph), the in-degree (number of connecting edges), and the page rank (importance score of a node within a directed graph) of nodes were computed.

Destination websites offering products for sale in the national data set were categorized as follows: legitimate online pharmacies, illegal medicine retailers (rogue online pharmacies), or dietary supplement seller (nonpharmacy web shops). Destination website categories were not defined for EU countries, so only links with redirection to illegal online sellers were documented regarding the international data set. Destination website legitimacy was checked at LegitScript [24] and categorized as approved, unlicensed, or rogue (illegitimate). The estimated number of monthly unique visitors of the root domain for all regions at the time of evaluation is provided by SEMrush traffic analytics [25].

Data were analyzed using SPSS Statistics 26 for Windows (IBM Corp.) and MS Excel (Microsoft Inc.).

### Ethical Considerations

There were no ethical issues, as only publicly available data obtained from SEs and websites were documented and evaluated. Furthermore, no customer or personal data were measured, recorded, or stored in this study.

## Results

### Compromised Websites Among SERPs of Medications for Treating Erectile Dysfunction in Hungary Between 2019 and 2021

The results show that during our 3-year observation period, there were no legitimate internet pharmacy websites among the evaluated SERPs. A decrease in the number of compromised sites linking visitors to illegitimate medicine sellers has been observed during our study period, while inaccessible broken links have increased. Similarly, the number of national rogue online pharmacies has increased in SERs up through 2021. All active ingredients have been affected by poisoning, with avanafil showing a somewhat diminished prevalence (Table 1).

**Table 1:** Top 20 search engine results page link categories for 4 erectile dysfunction medications.

Link category		August 2019, n (%)	October 2019, n (%)	August 2020, n (%)	October 2021, n (%)
Legitimate online pharmacy (n=80)^a^		0 (0)	0 (0)	0 (0)	0 (0)
National illegal medicinal product seller (n=80)		8 (10)	12 (15)	16 (20)	34 (43)
**International illegal medicinal product vendor via compromised site and redirection (active; n=80)**		43 (54)	33 (41)	25 (31)	4 (5)
	Avanafil (n=20)	9 (45)	5 (25)	3 (15)	0 (0)
	Sildenafil (n=20)	12 (60)	9 (45)	6 (30)	1 (5)
	Tadalafil (n=20)	12 (60)	9 (45)	8 (40)	1 (5)
	Vardenafil (n=20)	10 (50)	10 (50)	8 (40)	2 (10)
Compromised site without redirection (n=80)		5 (6)	3 (4)	1 (1)	0 (0)
Not accessible (eg, 404) at the time of evaluation (n=80)		2 (3)	7 (9)	9 (11)	15 (19)
Dietary supplement web shop (n=80)		9 (11)	10 (13)	14 (18)	8 (10)
Other sites not offering products for sale (n=80)		13 (16)	15 (19)	15 (19)	19 (24)

^a^According to national regulations, legitimate online pharmacies in Hungary cannot offer prescription medications—including oral medications for erectile dysfunction—via the internet.

Although most of the compromised websites were “true redirects” transferring individuals to international online sellers, we occasionally came across hacked sites without redirection. For example, in these cases, the rogue online pharmacy was operating under a subpage of the hacked domain, or the medication-related text was filled with keywords and links (so-called keyword stuffing and link building), indicating “black-hat” SEO techniques.

Such pages are likely to rank higher in search engines and develop redirects as time passes. In other instances, the web page we were looking for did not exist on the website’s server. Pages not accessible (eg, 404 error) at the time of evaluation could be related to website administrators identifying the malicious redirect code inserted into a website. According to our observation, hacking is followed by the malicious redirection life cycle, which consists of future (inactive pages ready to become active), active, and finally inactive stages.

**Figure 2:**
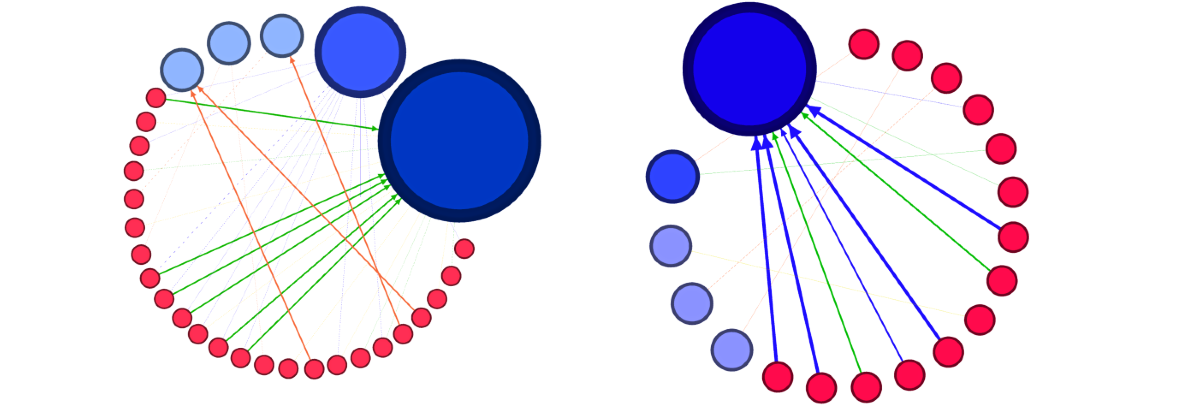
Visual graph of SERP links of compromised websites and illegal online medicine vendors accessed via search redirection attack visited in August 2019 (left) and August 2020 (right). SERP: search engine results page.

The complexity of the graphs decreased (the average degree changed from 1.17 to 0.667), between August 2019 and October 2021 (Figure 2). A majority (11/14, 79%) of the evaluated online pharmacies were categorized as rogue by LegitScript. We identified 5 destination online pharmacy websites in the link network at each evaluation date, except for October 2021. Initially, destination domains (eg, acs-pharmacy.com and evo-pharmacy.com) received numerous incoming links from SERs and played a central role in the network. By the end of the 3-year evaluation period, illegal pharmacy websites in-degree and page rank values underwent substantial reduction (Table 2). Website traffic analytics by SEMrush indicated a high number of monthly visitors (range 370-155,400) for important nodes with high page-rank values within the graph. This value illustrates the destination site’s global visitor count in the given month of evaluation.

**Table 2:** Graph statistics, legitimacy rating, and traffic history regarding referred illegal medicine vendors for Hungarian erectile dysfunction medication search queries.

Domain accessed following search redirection attack	Date	In-degree^a^	Page rank^b^	Legitimacy rating (LegitScript)	Number of unique visitors per month (SEMrush)^c^
acs-pharmacy.com	August 2019	16	0.209	Rogue^d^	155,400
acs-pharmacy.com	October 2019	16	0.332	Rogue	117,000
1-pharm.com	August 2019	12	0.140	Rogue	11,000
specialmedassortment.com	August 2019	2	0.054	Rogue	3600
myworldpharma.com	August 2019	2	0.054	Not in database	4000
pharmpillsonline.com	August 2019	2	0.054	Rogue	800
herbsandmeds.com	October 2019	2	0.061	Rogue	5200
pharmrx-1.com	October 2019	2	0.051	Rogue	6500
cheap-pharma.com	October 2019	1	0.042	Rogue	5100
big-pharmacy.com	October 2019	1	0.032	Rogue	15,600
evo-pharmacy.com	August 2020	9	0.279	Rogue	83,400
evo-pharmacy.com	October 2021	2	0.574	Rogue	30,400
eu-pharm.de	August 2020	2	0.087	Not in database	370
ezshopremedieshere.com	August 2020	1	0.059	Not in database	Not in database
canadarx24h.com	August 2020	1	0.059	Rogue	5200
medsalltheworld.com	August 2020	1	0.059	Rogue	3100

^a^In-degree value shows the number of links adjacent to a domain.

^b^The page rank algorithm measures the importance of each node within the graph.

^c^The estimated number of monthly unique visitors of the root domain for all regions at the time (month) of evaluation provided by SEMrush traffic analytics.

^d^Rogue: online pharmacy website engaged in illegal activity; a rating determined by LegitScript.

### International Relevance of Compromised SERPs in Europe 2020

A total of 1920 search results were evaluated in November 2020, in accordance with the results of the aforementioned 4 APIs listed in the top 40 results on the SERP pages throughout 12 European countries. Of those, 380 (19.79%) search query results were compromised, with a majority (n=342, 90%) of the links of the 230 infected source domains redirecting individuals to 73 international illegal medicinal product vendors. The remaining SER links were leading to compromised sites without redirection (6/380, 1.6%) or not accessible web pages/sites (32/380, 8.4%). Descriptive graph statistics of the international data set, website legitimacy category, and traffic history regarding destination online pharmacies with at least five referring links are depicted in Table 3.

**Figure 3:**
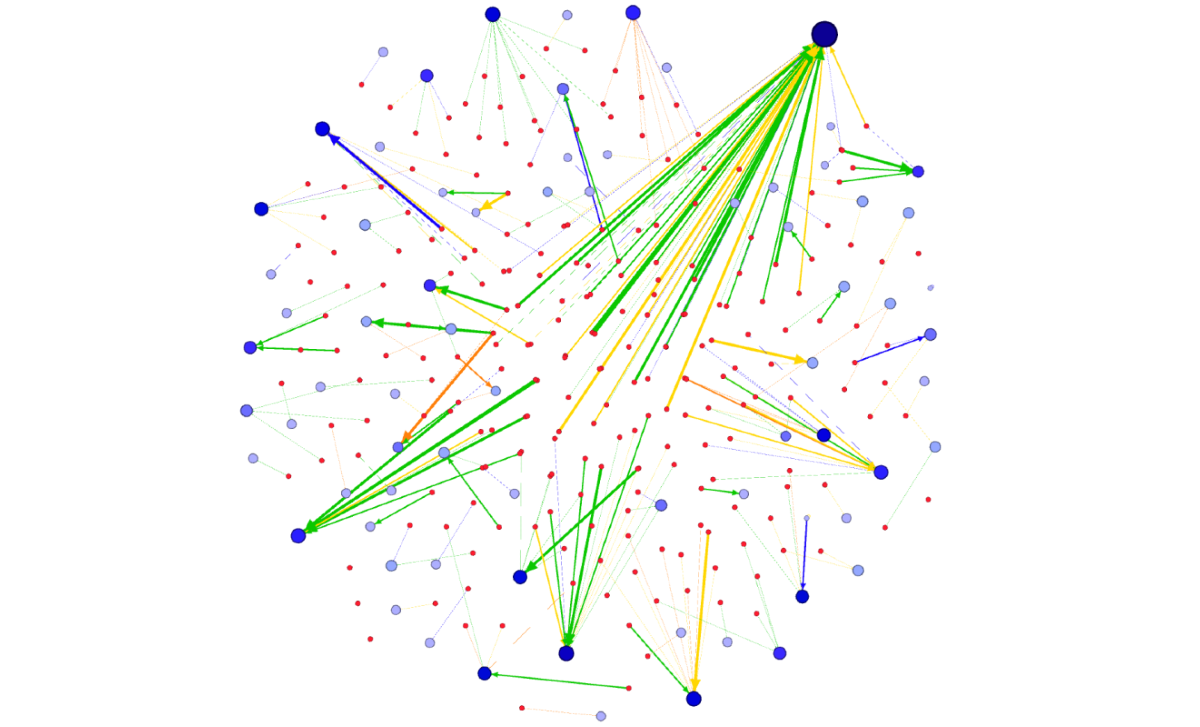
Graph of compromised websites (n=230) and illegal online medicine vendors (n=73) accessed via search redirection attack in 12 European countries visited in November 2020. Node size—represented by circles—illustrate the in-degree property of a domain in the graph. Small red nodes show compromised website domains in SERs and destination websites are labeled with blue. The edge—representing links—are colored based on the API name used in search queries (blue for sildenafil, green for vardenafil, yellow for tadalafil, and orange for avanafil). API: active pharmaceutical ingredient; SER: search engine result.

The most influential destination domain in the international redirection graph was “ezshopremedieshere.com,” with 79 referring links from search queries in most (8/12, 66%) of the evaluated European countries, and 61,400 unique global visitors in November 2020. Although several destination websites had numerous incoming links, the average in-degree value was 1.11, as most nodes had only 1 (30/79, 38%) or 2 (12/79, 15%) compromised referrals from search engines (Figure 3). The number of monthly global visitors per domain was the highest for “forecastarrays.us,” “cheapshopmed.com,” and “haiyuanpenguan.com,” attaining 566,100, 135,100, and 128,300 visitors, respectively, according to SEMrush traffic analytics. Interestingly, these high-traffic domains had only a small number (1-3) of incoming links from SERs and only 1 European country was affected in each case (Finland, Estonia, and Croatia, respectively). The “cheapshopmed.com” domain is a rogue online pharmacy in the LegitScript database. However, the “forecastarrays.us” and “haiyuanpenguan.com” domains contain compromised pages, including their intended content, and they can be accessed after redirection with an embedded online pharmacy content, so the visitor count of these domains is likely to include nonmedicinal purchase intention also. Website traffic estimation was available for 40 destination domains, with 35 having pharmacy-specific domain names (including terms, such as Rx, pharm, meds, pills). These 35 active online pharmacy domains, accessible from 12 European countries via compromised links in search engine queries, included a total of 473,118 unique visitors during November 2020.

**Table 3:** Graph statistics, legitimacy rating, and traffic history regarding selected referred illegal medicine vendors for erectile dysfunction medication search queries in 12 European countries (November 2020).

Domain accessed following search redirection attack	In-degree	Page rank	Countries affected	Legitimacy rating (LegitScript)	Number of unique visitors per month (SEMrush)
ezshopremedieshere.com	79	0.080	Croatia, Estonia, France, Greece, Hungary, Italy, Spain, Sweden	Not in database	61,400
evo-pharmacy.com	20	0.017	Hungary	Rogue	Not in database
rx-qualityshop.com	19	0.023	Croatia, Estonia, Finland, Romania, Sweden	Rogue	Not in database
your-meds-store.com	14	0.013	Croatia, Estonia, Finland, Greece, Italy, Romania, Spain	Rogue	4600
onlinepharmacyhub.com	13	0.018	Croatia, UK, Estonia, Romania	Not in database	2300
overnightpharm.com	11	0.015	UK, Estonia, France, Italy, Spain, Sweden	Rogue	321
rx-24-online.com	10	0.018	UK, Sweden	Rogue	Not in database
hot-med.com	9	0.017	Estonia, Spain	Rogue	21,500
usamedicineget.com	8	0.005	Croatia, Estonia, Romania	Rogue	5000
igohealth365.com	8	0.012	UK, France, Italy, Spain	Rogue	Not in database
qualitypillsprovider.com	7	0.007	Hungary, Spain, Sweden	Rogue	519
meds-store-24h.com	7	0.010	Finland, Greece, Italy, Spain	Rogue	7800
pills-group.com	6	0.010	Italy	Not in database	Not in database
vipcanadianstore.com	6	0.008	France, Italy, Sweden	Rogue	Not in database
online-secure-shop24h.com	6	0.009	Bulgaria, Greece, Italy, Spain	Rogue	8400

**Figure 4:**
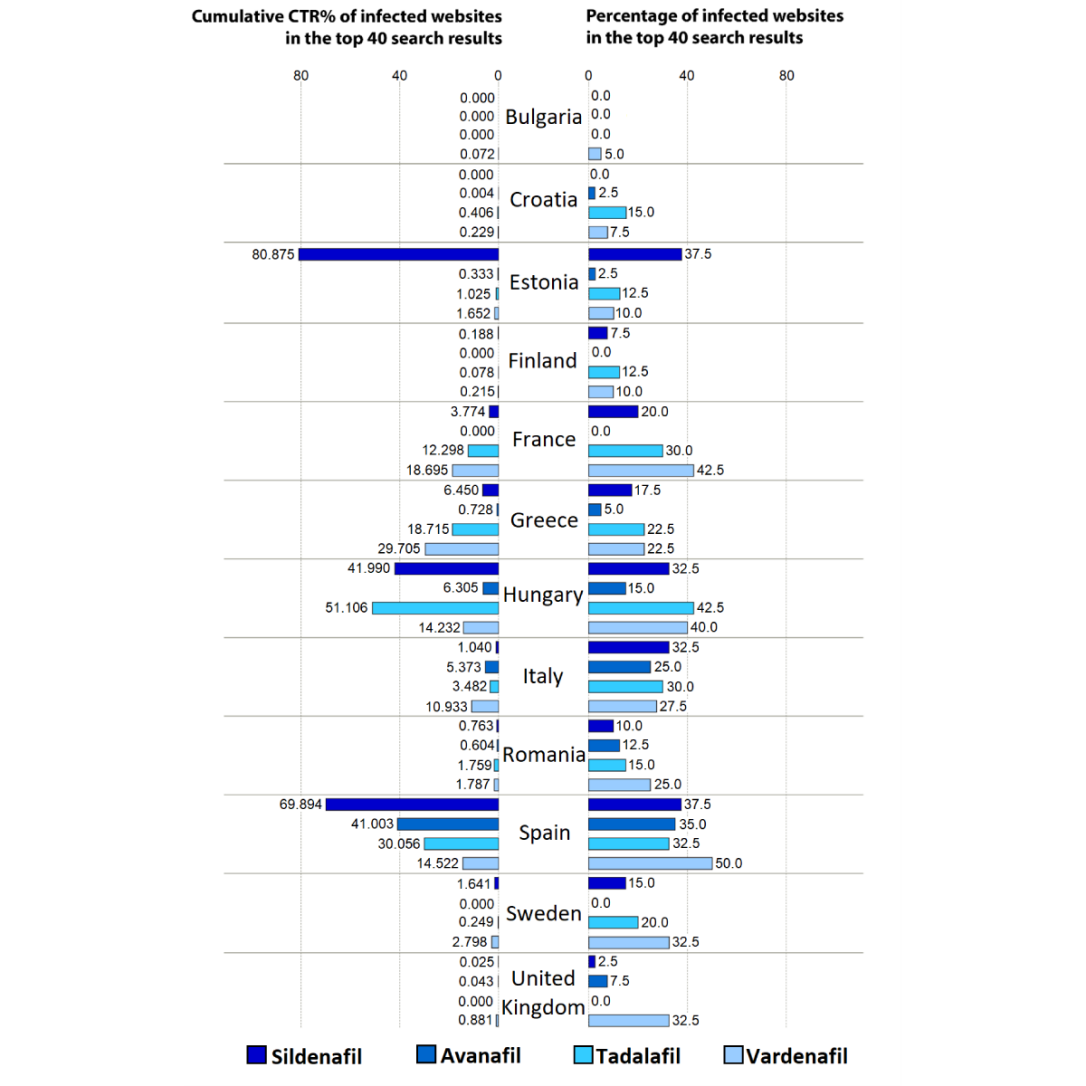
Cumulative click-through rate (CTR) prevalence of redirection links within search engine result pages leading to illegitimate online pharmacy websites search queries in 12 European countries.

The EU countries are affected differently by redirection links within SERPs, leading to illegitimate online pharmacy websites (Figure 4). In the “Methods” section, we proposed 2 metrics to illustrate the magnitude of the problem manifested throughout European countries. The proportion of the hacked pages as a percentage of the total search query results and the cumulative CTR percentages were calculated to illustrate the issue of the compromised websites in a complex manner in each country’s SERP. It is important to view cumulative CTR and the number of compromised websites as both unique and complementary factors. To state an example, if a country’s SERP has several websites lower down the list, the cumulative CTR will be minimal. However, these websites pose a potential risk of rising surreptitiously quickly through the ranks and gaining higher CTRs.

Compromised links redirecting to active online pharmacies were present in search query results of all evaluated countries. The prevalence of compromised links in national SERs was the highest in Spain (62/160, 38.8%), Hungary (52/160, 32.5%), Italy (46/160, 28.8%), and France (37/160, 23.1%), whereas it was the lowest in Finland (12/160, 7.5%), Croatia (10/160, 6.3%), and Bulgaria (2/160, 1.3%). Cumulative CTR values computed for APIs indicated the highest potential impact and danger of search engine redirection attacks for avanafil in Spain (41.0%), sildenafil in Estonia (80.9%), tadalafil in Hungary (51.1%), and vardenafil in Greece (29.7%). Prevalence and cumulative CTR metrics were relatively high for all APIs in Hungary and Spain, indicating a larger number of infected SER links with relatively high-ranking positions in search queries. Accordingly, consumers searching for erectile dysfunction medications online are more likely affected by online medicine purchase opportunities presented by illegal online pharmacies applying search engine redirection attack as a marketing technique in these countries. Although SERs in Romania, Finland, and Greece contain a substantial number of compromised links, because of low rankings, the cumulative CTR vales are low, indicating that consumers are less likely to click on compromised links leading to the destination illegal online pharmacy websites. The complete redirection network is illustrated in Figure 3.

Hacked websites are not specialized in active ingredients and target domains. Of the observed 230 infected source domains, many (n=65, 28.3%) promote various APIs. Although the majority (160/230, 69.6%) of source infections drive traffic to a single destination, many redirect individuals to various online pharmacy websites (range 1-6; mean 1.49 redirection links of independent destination domains).

## Discussion

### Principal Findings

The evolution of online advertising methods and specialization have led to the development of affiliate networks, an established method for legitimate merchants in which sponsors pay a commission to advertisers delivering traffic to their websites. Unfortunately, illegal online pharmacies are also a typical example of affiliate networks and search engine poisoning is a tool linked to affiliates to convert visitors from search engines. A robust number of independent affiliates, acting as advertisers or traffic brokers, received high (30%-40%) commissions for promoting illegal medication vendors and delivering traffic to the sponsor websites in which medications are sold to customers [14]. This affiliate program business model has numerous advantages for its participants. Sponsors (destination illegal pharmacy websites) do not have to heavily invest in marketing campaigns. Even more advantageous is that they free themselves from direct exposure to the criminal risks associated with large-scale advertising. Affiliates generate sales for sponsors by only focusing on attracting customers without developing web shops, customer service, etc. Online pharmaceutical sales are one of the oldest and largest affiliate program markets, with an estimated turnover of 500,000-600,000 customers, 700,000 billed orders, and US $73,000,000-85,000,000 revenue per 3-year period (2007-2010) analyzed by McCoy et al [14] referencing 2 major affiliate networks (Glavmed and SpamIt). By evaluating the change of new customer acquisitions, the authors concluded that affiliate programs attract new customers at a steady rate (approximately 3300/week). Thus, the market of counterfeit pharmaceuticals was not saturated, suggesting latent customer demand [14]. Furthermore, the same data set provides evidence for customer loyalty and satisfaction regarding online pharmacies, as repeat purchases constitute more than 20% of overall revenue. Our previous findings also indicate that a vast number of online pharmacies operate illegally and offer medicines to buyers in the long run [10].

It has been estimated that the number of men experiencing erectile dysfunction worldwide can reach 332 million by 2025 [19]. Erectile dysfunction medications containing PDE5 inhibitors are highly prone to falsification with proven potential health risk for patients. Analytical investigation of these products often shows the presence of dangerous excipients of nonpharmaceutical origin or quality, more than 1 undeclared PDE5s, and active ingredient amounts higher than declared values often surpassing the maximum therapeutic dose [5]. Previous research [26] regarding patient safety risks assessment of the online market of medicinal products revealed that Google search results include several suspicious links. By clicking on these SERs, the visitor is apparently redirected to an unlicensed drug distribution page by initially clicking on the link of a legitimate, yet irrelevant domain. This unfair online marketing of search redirection attack is thought to play a decisive role in the illegal internet pharmaceutical marketplace. Although search engine redirection attacks leading visitors to illegal online pharmacy networks have been previously published [9,16], we did not find relevant publications in medical informatics journals during the past decade. Admittedly, search engine redirection attacks are not limited to Google, the most popular search engine. The same phenomena could be identified in Microsoft Bing and Yahoo!. Seemingly, this unsolved issue has sunk into oblivion. This study was aimed to describe, map, and highlight its national and international significance.

Nearly half of search results were redirecting individuals to illicit medicine vendor sites during our national results obtained in 2019, with compromised websites being dominant in SERPs. This finding correlates with a previous study by Leontiadis et al [16], highlighting how redirections constitute the most significant proportion of results for the query set implemented in this study. Although the prevalence of compromised links in SERs and the complexity of the graphs have decreased in our national data set between August 2019 and October 2021, the danger has not dissipated. Consumers searching for ivermectin during the COVID-19 pandemic were more likely to find links redirecting to illegal medicine retailers that represent 73.3% of SER links within the first 30 search results in Google in March 2021 [26]. Despite the attempts to prevent this “black hat” SEO technique proposed a decade ago, limited success can be observed [9], and we are facing a constant issue that has not been solved for a relatively lengthy period.

Our international search query data set obtained from a representative sample of SERs among 12 European countries illustrates the international significance of search engine poisoning. All evaluated countries are affected, as at least one of four active ingredients for the treatment of erectile dysfunction was offered for sale via compromised links. The overall prevalence of hacked links in SERs was highest among Spain, Hungary, Italy, and France. Among 1920 manually evaluated links, we documented 380 compromised results from a total of 230 websites (domains) leading to 73 illegal online medicine vendors. The majority of these illegal online pharmacies (41/73, 56%) received only 1 or 2 compromised links. Meanwhile, the top 3 domains with the highest in-degree property received more than one-third of all incoming links. These findings support earlier studies stating that illicit advertising business is dominated by only a handful of big-league players [16].

An important implication regarding our findings is that search-redirection attackers use a complex system with potentially vulnerable elements to convert traffic to their illegitimate destination websites. We conclude that such practices can be disrupted by various stakeholders in a number of ways (Textbox 1).

Most likely, if any 1 or more than 1 of the aforesaid measures are considered, the redirection network collapses, and infected source websites will not appear, nor will they rank high in the search results. Lastly, they will not actively redirect to illegitimate online pharmacy domains.

A common feature of the aforesaid measures is the undisrupted continuity of the system, as it most likely requires time to build up such a complex network among numerous stakeholders. Findings of previously published literature suggest that the median survival time of a source infection is 19 days; however, some claim a lot lengthier time (17% of infections lasted at least six months, while 8% survived for more than 1 year) [16]. Our findings also corroborate this, as 4 compromised pages in our national data set remained in the top 20 results for more than 2 years, between August 2019 and October 2021.

**Possible solutions to overcome search-redirection poisoning redirecting to illegal internet pharmacies.**

- Search providers and authorities can identify compromised links by monitoring popular medicinal product–related search terms (eg, brand or active ingredient name of prescription medications), as infected websites contain numerous relevant keywords and links to rank high in search engine results pages (SERPs) for popular queries and to publicize themselves.
- In addition to manual evaluation of SERPs, previously published link-based and content-based algorithms as well as tailor-made automatic detection and classification engines can be used as benchmarks in the effective identification of pharma scam campaigns [27].
- Search engine providers play a decisive role in monitoring and moderating SERPs. Without their dedicated and comprehensive effort, SERPs may never be free of compromised links leading to illegal online pharmacy networks. Automated URL-based classification methods, similar to deSEO [28] proposed in 2011, can only be applied if search engine providers provide search query logs to authority or academic parties.
- If operators fail to identify the infection, compromised websites remain among the top results and maintain the functionality of redirecting. Consequently, the operators of vulnerable legitimate domains should be notified so that they can take action to improve content management system security and remove hacked pages.
- The intermediate redirection chain elements need to remain operational for effective redirection and search engine optimization, so when the webmaster removes the infection triggering the redirection, or any intermediary page, the redirection chain ceases to function.
- The destination illegitimate online pharmacies must stay online to remain operational. Therefore, drug authorities and law enforcement agencies can shut down final destination domains of rogue online pharmacies with a high number of incoming links and unique visitors.

As the number of infected websites appearing in SERPs and all other compromised websites within the redirection chain is considerably high and the number of destination websites are relatively low, it is reasonable to take measures against the latter by shutting down websites and domains. However, the efficacy of this intervention does not seem to be efficient enough, considering the fact that the Operation Pangea coordinated by Interpol has taken down more than 150,000 websites between 2008 and 2020. Despite this large-scale removal, an extremely large number of links (113,020 websites and online marketplaces) were subsequently closed down in 2021 [29,30], demonstrating the substantial scale and recurrence of this issue, which remains unresolved.

### Limitations

Admittedly, our study bears several limitations, for instance, the search query results of only 1 search engine have been summarized; however, we believe that the validity of our methodology can be explained by the dominant market share of the search engine. Furthermore, as opposed to brand-name queries, API-based search may offer varied results; however, Google’s complex algorithm is likely to provide results for related searches. API was used because our aim was to find all relevant websites, regardless of their original and generic names, varying from country to country, including unapproved generics and falsified medicines. Legitimacy of all final destination websites cannot be evaluated objectively, as there is no reliable database to evaluate all websites. However, we assumed all online medicine vendors using search engine redirection attack to attract customers and offer prescription medicines for sale most likely bear malicious intent and can be categorized as illegitimate online pharmacies.

In conclusion, our results illustrate that the phenomena of search engine poisoning have been persistent during the past decade and affiliate networks linked to illegitimate online pharmacies are flourishing. This supports the presumption that uncoordinated interventions aiming at ceasing illicit medicinal online purchases by authorities and individual stakeholders are not yet sufficient. It is a problem that has not been solved for more than a decade. Importantly, uncontrolled illegal sale of medications has many unfavorable consequences for the health of consumers and the safety of the pharmaceutical supply chain. Detecting and eliminating malicious links promoting illegal online pharmacies in search engines are of great importance with regard to cybersecurity and patient safety.

## Abbreviations

API: active pharmaceutical ingredient
CTR: click-through rate
EU: European Union
PDE5: phosphodiesterase type 5
SEO: search engine optimization
SER: search engine result
SERP: search engine results page

## References

[1] Eurostat (2022). Internet purchases - goods or services (2020 onwards) Internet. Eurostat.

[2] Fittler A, Vida RG, Káplár Mátyás, Botz L (2018). Consumers Turning to the Internet Pharmacy Market: Cross-Sectional Study on the Frequency and Attitudes of Hungarian Patients Purchasing Medications Online. J Med Internet Res.

[3] Orizio G, Merla A, Schulz PJ, Gelatti U (2011). Quality of online pharmacies and websites selling prescription drugs: a systematic review. J Med Internet Res.

[4] Fittler A, Vida RG, Rádics Valter, Botz L (2018). A challenge for healthcare but just another opportunity for illegitimate online sellers: Dubious market of shortage oncology drugs. PLoS One.

[5] Gaudiano MC, Manna L, Rodomonte AL, Bartolomei M, Bertocchi P, Gallinella B, Antoniella E, Muleri N, Civitelli G, Alimonti S, Romanini L, Rufini L, Valvo L (2012). A survey on illegal and counterfeit medicines for the treatment of erectile dysfunctions in Italy. J Sex Med.

[6] Blackstone E, Fuhr J, Pociask S (2014). The health and economic effects of counterfeit drugs. Am Health Drug Benefits.

[7] Mackey TK, Nayyar G (2016). Digital danger: a review of the global public health, patient safety and cybersecurity threats posed by illicit online pharmacies. Br Med Bull.

[8] Goodwin D 71 Mind-Blowing Search Engine Optimization Stats Internet. searchenginejournal.

[9] Lu L, Perdisci R, Lee W (2011). SURF: detecting and measuring search poisoning. CCS '11: Proceedings of the 18th ACM conference on Computer and communications security.

[10] Fittler A, Bősze G, Botz L (2013). Evaluating aspects of online medication safety in long-term follow-up of 136 Internet pharmacies: illegal rogue online pharmacies flourish and are long-lived. J Med Internet Res.

[11] The Center for Safe Internet Pharmacies (2016). The Internet Pharmacy Market in 2016: Trends, Challenges and Opportunities. The Center for Safe Internet Pharmacies.

[12] National Association of Boards of Pharmacy (2017). Internet Drug Outlet Identification Program: Progress Report for State and Federal Regulators Internet. National Association of Boards of Pharmacy.

[13] Shahzad A, Mahdin H, Mohd N (2020). An Improved Framework for Content-based Spamdexing Detection. IJACSA.

[14] McCoy D, Pitsillidis A, Jordan G, Weaver N, Kreibich C, Krebs B, Voelker G, Savage S, Levchenko K (2012). PharmaLeaks: Understanding the business of online pharmaceutical affiliate programs. Security'12: Proceedings of the 21st USENIX conference on Security symposium.

[15] Leontiadis N, Moore T, Christin N (2011). Measuring and Analyzing Search-Redirection Attacks in the Illicit Online Prescription Drug Trade. SEC'11: Proceedings of the 20th USENIX conference on Security.

[16] Leontiadis N, Moore T, Christin N (2014). A Nearly Four-Year Longitudinal Study of Search-Engine Poisoning. CCS '14: Proceedings of the 2014 ACM SIGSAC Conference on Computer and Communications Security.

[17] Wang D, Savage S, Voelker G (2011). Cloak and dagger: dynamics of web search cloaking. CCS '11: Proceedings of the 18th ACM conference on Computer and communications security.

[18] Madeira CR, Tonin FS, Fachi MM, Borba HH, Ferreira VL, Leonart LP, Bonetti AF, Moritz RP, Trindade ACLB, Gonçalves Alan G, Fernandez-Llimos F, Pontarolo R (2021). Efficacy and safety of oral phosphodiesterase 5 inhibitors for erectile dysfunction: a network meta-analysis and multicriteria decision analysis. World J Urol.

[19] Ayta IA, McKinlay JB, Krane RJ (1999). The likely worldwide increase in erectile dysfunction between 1995 and 2025 and some possible policy consequences. BJU Int.

[20] Ahmed J, Modica de Mohac L, Mackey TK, Raimi-Abraham BT (2022). A critical review on the availability of substandard and falsified medicines online: Incidence, challenges and perspectives. J Med Access.

[21] Orizio G, Schulz P, Domenighini S, Caimi L, Rosati C, Rubinelli S, Gelatti U (2009). Cyberdrugs: a cross-sectional study of online pharmacies characteristics. Eur J Public Health.

[22] Beus J (2020). Why (almost) everything you knew about Google CTR is no longer valid Internet. sistrix.

[23] Gephi - The Open Graph Viz Platform. Gephi.

[24] LegitScript Website Status Checker Internet. LegitScript.

[25] Traffic Analytics Internet. SEMrush.

[26] Fittler A, Adeniye L, Katz Z, Bella R (2021). Effect of Infodemic Regarding the Illegal Sale of Medications on the Internet: Evaluation of Demand and Online Availability of Ivermectin during the COVID-19 Pandemic. Int J Environ Res Public Health.

[27] Corona I, Contini M, Ariu D, Giacinto G, Roli F, Lund M, Marinelli G (2015). PharmaGuard: Automatic identification of illegal search-indexed online pharmacies.

[28] John PJ, Yu F, Xie Y, Krishnamurthy A, Abadi M (2011). deSEO: Combating Search-Result Poisoning. https://www.usenix.org/legacy/events/sec11/tech/full_papers/John.pdf.

[29] Lee KS, Yee SM, Zaidi STR, Patel RP, Yang Q, Al-Worafi YM, Ming LC (2017). Combating Sale of Counterfeit and Falsified Medicines Online: A Losing Battle. Front Pharmacol.

[30] (2021). Thousands of fake online pharmacies shut down in INTERPOL operation. Interpol.

